# Incidence and factors associated with postoperative delirium in patients undergoing transurethral resection of bladder tumor

**DOI:** 10.1186/s40981-022-00497-5

**Published:** 2022-01-22

**Authors:** Shohei Nakatani, Mitsuru Ida, Xiaoying Wang, Yusuke Naito, Masahiko Kawaguchi

**Affiliations:** Department of Anesthesiology, Nara Medical University, Kashihara, Nara, 634-8522 Japan

**Keywords:** Age, Body mass index, General anesthesia, Postoperative delirium, TUR-BT

## Abstract

**Background:**

Postoperative delirium is an important complication after surgery, including urological surgery. This study evaluated the incidence of postoperative delirium and its associated factors after transurethral resection of bladder tumor in adult patients.

**Methods:**

Patients aged ≥20 years who underwent elective transurethral resection of bladder tumor under general anesthesia from April 2016 to November 2020 were included. Patient demographic and intraoperative data, including the administration of 5-aminolevulinic acid and hypotension, defined as a mean arterial pressure value < 60 mmHg, were evaluated. The primary outcome was the incidence of postoperative delirium assessed using a chart-based method. The factors associated with postoperative delirium were explored using multiple logistic regression analysis. Postoperative lengths of stay between patients with and without postoperative delirium were compared using the Mann–Whitney *U*-test.

**Results:**

Of 324 eligible patients with a median age of 76, 26 patients experienced postoperative delirium, with an incidence rate of 8.0% (95% confidence interval, 5.06–10.9). Age (odds ratio 1.13, 95% confidence interval 1.05–1.22, *p* = 0.001) and body mass index (odds ratio 0.83, 95% confidence interval 0.71–0.97, *p* = 0.02) were associated with postoperative delirium. Postoperative length of stay between patients with or without postoperative delirium was not significantly different (6 vs 6 days, *p* = 0.18).

**Conclusions:**

The incidence of postoperative delirium after transurethral resection of bladder tumor under general anesthesia in this study was 8.0%. Older age and low body mass index were associated with development of postoperative delirium.

**Supplementary Information:**

The online version contains supplementary material available at 10.1186/s40981-022-00497-5.

## Background

Postoperative delirium (POD) is one of the most common postoperative complications and negatively impacts the length of postoperative hospital stay and long-term mortality [1, 2]. In urological surgeries, the incidence of POD ranges from 3.4 to 46% depending on the surgical technique and assessment method [3–6], and some factors, including older age and longer duration of surgery, are associated with POD [3, 5, 7].

Transurethral resection of bladder tumor (TUR-BT) is a standard surgical procedure for bladder tumors. At the first glance, TUR-BT is expected to have a lower incidence of POD considering its shorter surgical duration and minimally invasive approach compared with other major urological procedures such as radical cystectomy and nephrectomy. These advantages are also the reasons why older patients are more likely to undergo TUR-BT; because advanced age is a risk factor for POD, the incidence of POD after TUR-BT may indeed be higher than expected. However, little evidence is currently available regarding POD after TUR-BT. Hypotension often occurs during anesthesia, but the impact of intraoperative hypotension on POD has not been fully investigated in non-cardiac surgery [8]. Additionally, TUR-BT using 5-aminolevulinic acid (5-ALA) has become a common procedure to facilitate the identification of an accurate resection range and this is associated with a risk of intraoperative hypotension [8].

To evaluate the incidence of POD following TUR-BT and its associated factors, we performed a secondary analysis using data from a study that showed that preoperative oral 5-ALA was associated with intraoperative hypotension in patients undergoing TUR-BT [9].

## Methods

### Ethical approval

The original study and this secondary analysis were approved by Nara Medical University Institutional Review Board, Kashihara, Nara, Japan (Number 2904). The need for informed consent was waived owing to the retrospective nature of this study.

### Patient cohort

Patients aged 20 years or older who underwent elective TUR-BT for bladder tumors under general anesthesia from April 2016 to November 2020 at our tertiary teaching hospital were eligible for enrolment in our original study. Patients with a preoperative diagnosis of dementia and who required additional procedures in addition to TUR-BT were excluded. Patients with missing data were also excluded from the analysis.

### Data collection

Age, sex, body mass index, preexisting medical conditions (hypertension, ischemic heart disease, symptomatic stroke, diabetes), pulmonary function (normal, obstructive lung disease, restrictive lung disease), plasma albumin, plasma creatinine, American Society of Anesthesiologists-Physical Status (ASA-PS), and preoperative administration of 5-ALA were obtained from the electric medical records. We defined patients taking antihypertensive drugs and oral diabetic drugs or injectable insulin as patients with hypertension and diabetes mellitus, respectively. Patients were considered to have obstructive lung disease if their forced expiratory volume 1.0 (s) % was <70%; restrictive lung disease was defined as a vital capacity of <80%. Patients with ischemic heart disease were defined as those having a history of coronary artery bypass graft and/or percutaneous coronary intervention. Intraoperative data including anesthetics (inhalation agents or propofol), intraoperative hypotension, and duration of anesthesia were also assessed. Intraoperative hypotension was defined as a mean arterial pressure value < 60 mmHg, in accordance with a recent review [10].

### Outcome

The primary outcome for this study was the incidence of POD and its associated factors occurring during the first 7 postoperative days or up to the day of discharge; these data were obtained using a chart-based method for the prediction of delirium [11]. This method, which has been widely used to identify delirium after anesthesia and during intensive care, allows for the assessment of delirium during the evaluation period, including overnight [12–14]. The secondary outcome was the difference in postoperative length of stay categorized based on POD.

### Statistical analysis

Data are presented as median [first quartile, third quartile] or *n* (%). Univariate analysis was performed using Fisher’s exact test or Mann–Whitney *U*-test for categorical or continuous variables, respectively. Multiple logistic regression analysis with all explanatory variables was used to identify variables associated with the primary outcome. In multiple logistic regression analysis, ASA-PS was divided into two groups, namely, ASA-PS 1 and 2 or ASA-PS 3; then, ASA-PS 3 was included as one of the covariates. Furthermore, the postoperative length of stay was compared between patients with POD or without POD using the Mann–Whitney *U*-test. *p* < 0.05 was considered statistically significant.

Sample size was not calculated due to the nature of a secondary analysis and was based on our original study; thus, the incidence of POD is expressed as a percentage, with the 95% confidence interval (CI) calculated using the Wald method. The number of cases ≥10 times the explanatory variable for the number of cases with few outcomes is recommended to perform the logistic regression analysis. In this study, of the covariates collected in our original study, only factors considered to be strongly associated with POD were analyzed; however, the number of these covariates exceed the recommended number. Thus, not only results of the logistic regression analysis, such as odds ratios and *p* values, but also those of the Hosmer–Lemeshow test and AUC were analyzed. The Hosmer–Lemeshow test was used to test the calibration of the model, and the area under the receiver operating characteristic curve (AUC) was computed as a descriptive tool for measuring model bias.

### Literature review

In 2020, a well-designed systematic review of postoperative delirium after urologic surgery was published [7]; however, few systematic reviews have evaluated POD after transurethral resection (TUR). Thus, in order to understand the current status of POD after TUR, we performed an additional search on July 11, 2021, using MEDLINE, Embase, PsycINFO, CINAHL, and Cochrane library to identify new studies published from January 23, 2020 (last day which the previous systematic review included in the search), to July 10, 2021, that evaluated POD after urological surgery. We then planned to extract studies focusing on TUR. Randomized controlled trials, prospective and retrospective observational studies, and systematic reviews with or without meta-analysis written in English were eligible for inclusion. The manuscript titles and abstracts were screened independently by two of the authors (SN and MI). Finally, inconsistencies were resolved by discussion. The full search strategy is presented in Supplemental Table 1.

## Results

Of 324 eligible patients, 26 patients experienced POD, with an incidence rate of 8.02% (95% *CI* 5.06–10.9). Table 1 shows the results of the univariate analysis. Patients who experienced POD were older; however, there were no differences in the rate of preoperative 5-ALA administration and intraoperative hypotension between patients who did and did not experience POD. As shown in Table 2, multiple logistic regression analysis revealed that age (odds ratio 1.13, 95% *CI* 1.05–1.22, *p* = 0.001) and body mass index (odds ratio 0.83, 95% *CI* 0.71–0.97, *p* = 0.023), which was not statistically significant in univariate analysis, were associated with the incidence of POD. The Hosmer–Lemeshow test did not reject a logistic regression model fit (*p* = 0.85). The explanatory model based on these variables had an area under the receiver operating characteristic curve of 0.79 (95% *CI*, 0.71–0.88). There was no statistical difference in the postoperative length of stay between patients with or without POD (6 [5, 8] vs. 6 [6, 8] days, *p* = 0.18).

**Table 1:** Patient demographics and intraoperative data of patients with and without postoperative delirium

	POD (−) (*n* = 298)	POD (+) (*n* = 26)	*p* value
Age (years)	75.0 [69.0, 80.0]	81.0 [76.0, 84.0]	0.0003
Sex: male	237 (79.5)	24 (92.3)	0.19
Body mass index (kg/m^2^)	23.5 [21.3, 25.8]	22.6 [19.8, 24.8]	0.062
Preexisting medical conditions			
Hypertension	169 (56.7)	14 (53.8)	0.83
Ischemic heart disease	266 (89.3)	21 (80.8)	0.19
Symptomatic stroke	43 (14.4)	5 (19.2)	0.56
Diabetes	87 (29.2)	7 (26.9)	1
Pulmonary function			0.16
Normal	182 (61.0)	12 (46.1)	
Obstructive lung disease	84 (28.2)	12 (46.2)	
Restrictive lung disease	32 (10.7)	2 (7.7)	
Plasma albumin (g/dL)	4.2 [4.0, 4.4]	4.1 [3.8, 4.3]	0.14
Plasma creatinine (mg/dL)	0.91 [0.76, 1.15]	0.89 [0.82, 1.09]	0.82
ASA-PS			0.79
1 or 2	54 (18.1)	5 (19.2)	
3	244 (81.9)	21 (80.8)	
5-ALA	141 (47.3)	12 (46.2)	1
Intraoperative data			
Anesthetics			0.37
Inhalation agents	284 (95.3)	24 (92.3)	
Propofol	14 (4.6)	2 (7.6)	
Intraoperative hypotension	49 (16.4)	4 (15.4)	1
Duration of anesthesia (min)	104 [84, 130]	120 [95, 144]	0.08

Median [first quartile, third quartile] or number (%)

*POD* postoperative delirium, *ASA-PS* American Society of Anesthesiologists physical status, *5-ALA* 5-amino levulinic acid

**Table 2:** Results of multiple logistic regression analysis for postoperative delirium

	Odds ratio	95% confidence interval	*p* value
Age (years)	1.13	1.05–1.22	0.001
Sex: male	0.21	0.04–1.14	0.072
Body mass index (kg/m^2^)	0.83	0.71–0.97	0.023
Preexisting medical conditions			
Hypertension	0.8	0.31–2.10	0.66
Ischemic heart disease	1.25	0.32–4.74	0.74
Symptomatic stroke	1.11	0.31–3.89	0.86
Diabetes	1.07	0.38–3.02	0.89
Pulmonary function			
Normal	1		
Obstructive lung disease	1.75	0.69–4.41	0.23
Restrictive lung disease	0.5	0.09–2.86	0.44
Plasma albumin (g/dL)	0.85	0.27–2.64	0.78
Plasma creatinine (mg/dL)	0.56	0.14–2.23	0.41
ASA-PS			
1 or 2	1		
3	0.68	0.17–2.7	0.58
5-ALA	1.03	0.39–2.71	0.95
Intraoperative data			
Anesthetics			
Inhalation agents	1		
Propofol	2.52	0.41–15.1	0.31
Intraoperative hypotension	0.88	0.23–3.32	0.85
Duration of anesthesia (min)	1.01	0.99–1.01	0.24

*ASA-PS* American Society of Anesthesiologists physical status, *5-ALA* 5-amino levulinic acid

### Literature review

The literature search yielded 270 papers. After a careful selection process (Preferred Reporting Items for Systematic Reviews and Meta-Analyses study flow diagram, Supplemental Figure 1), 19 papers, including six original studies and 13 systematic reviews, were selected as candidates. After a hand research of the references cited in previous systematic reviews, we screened an additional 49 original studies; then, of the 55 original studies, 39 were excluded. Of the remaining 16 studies, MI contacted the corresponding authors of all but three studies, in which surgical techniques were clear, in order to obtain accurate information. Two authors provided the details, one author stated his or her study had no patient undergoing TUR, and the other authors did not respond. Finally, five papers were included in our review, and the study characteristics are presented in Table 3. The mean age of patients included in all studies was >70 years, four studies included patients undergoing transurethral resection of the prostate (TUR-P) [3, 15, 17, 18], and only two studies assessed POD after TUR-BT [3, 16]. The incidence of POD ranged from 1.2 to 21.2% depending on the assessment tool of delirium. One study evaluated associated factors with POD, which included age and pain score[17].

**Table 3:** Overview of original studies assessing postoperative delirium after transurethral resection

Authors	Year	Number of patients			Surgical procedure	Anesthesia	POD metric	POD incidence	Associated factors with POD
		Total	TUR	Mean age (years)					
Tai et al. [15]	2015	485	485	71.2 (2.3)	TUR-P	Spinal	CAM	21.2% (103/485)	Not assessed using multivariate analysis
Sato et al. [16]	2016	215	39	73.8 (10)	TUR-BT	General and spinal	DSM-V	2.5% (1/39)	Not assessed specifically for patients who underwent TUR
Xue et al. [17]	2016	358	358	75.0 (6.3)	TUR-P	General and spinal	CAM	7.8% (28/358)	Age and pain score (multivariate analysis)
Braga et al. [18]	2019	55	55	71.8 (5.7)	TUR-P	No information was provided	CAM	5.4% (3/55)	Not assessed using multivariate analysis
Matsuki et al. [3]	2020	949	397	76.7 (6.9)	TUR-BT	General and spinal	ICDSC	1.2% (5/397)	Not assessed specifically for patients who underwent TUR
			86	73.7 (5.7)	TUR-P/PVP			4.6% (4/86)	
Nakatani et al. [8] (this study)	2021	324	324	74.6 (9.2)	TUR-BT	General	Chart-based method	8.0% (26/324)	Age and body mass index (multivariate analysis)

Mean (standard deviation) or number

*TUR* transurethral resection, *POD* postoperative delirium, *TUR-P* transurethral resection of the prostate, *CAM* confusion assessment method, *TUR-BT* transurethral resection of the bladder tumor, *TUL* transurethral ureterolithotripsy, *PVP* photoselective vaporization of the prostate, *DSM* Diagnostic and Statistical Manual of Mental Disorders, *ICDSC* Intensive Care Delirium Screening Checklist

## Discussion

The incidence of POD after TUR-BT was as high as 8.0%; however, there was no relationship between prolonged hospital stay and POD. Surprisingly, this incidence was comparable to the incidence of POD (7.3%) in patients aged ≥65 years who underwent elective abdominal surgery under general anesthesia in our hospital [14]. In addition to patient age, which is a well-known risk factor for POD, lower preoperative body mass index was found to be associated with POD risk.

Previous studies assessing POD using the Diagnostic and Statistical Manual of Mental Disorders V (DSM-V) and Intensive Care Delirium Screening Checklist (ICDSC) reported lower incidences of 2.5% and 1.2%, respectively [3, 16]. The DSM-V is not a screening tool but a diagnostic tool; thus, this lower incidence is reasonable. In contrast, the ICDSC and chart-based method, which are the assessment tools used in this study, are screening tools for delirium, but the ICDSC has lower sensitivity (66.7%) and specificity (78.1%) [19] than the chart-based method (sensitivity 74% and specificity 83%) [11]. This fact and the frequency of the screening of POD may have led to a higher incidence of POD in our study.

Regardless of the surgical technique, advanced age is among one of the most well-recognized non-modifiable risk factors for POD [20, 21], while preoperative low body mass index (i.e., low body weight) is a modifiable factor. Low body weight could reflect low skeletal muscle mass, also known as sarcopenia, which is a risk factor for POD [22, 23]. However, preoperative exercise training significantly contributes to increasing muscle mass [24]; therefore, our results provide the hypothesis whether increasing muscle mass can reduce the incidence of POD will be one of the most important topics for future research.

In this study, intraoperative hypotension occurred in 16.3% of patients. However, it did not affect the occurrence of POD. Previous studies focusing on the relationship between intraoperative hypotension and POD had significant heterogeneity, thus generating conflicting results regarding the association between hypotension and POD [8, 25, 26]. Furthermore, although the exact mechanism of delirium is unclear, neuro-inflammation may play an important role in its development [27]. Systemic hypotension can cause cerebral and cardiac hypoperfusion but not systemic inflammation, which may not be related to delirium; however, elucidating the mechanism underlying the association of hypotension and POD was beyond the scope of this study, and this should be explored in future studies.

Our study has some limitations. First, delirium was assessed using the chart-based method. The confusion assessment method is a standard delirium assessment tool, but it only provides information at the time of assessment. A chart-based method can provide the information regarding delirium during a hospital stay as long as delirium events are documented in the medical chart. Second, we only evaluated pre- and intraoperative data; thus, the results might be different if we had included postoperative data such as pain score and sleep quality. Additionally, irritation caused by a urethral catheter could be associated with POD. However, its association is expected to be difficult to be proven because patients who clearly complain of the urethral catheter recognize their situation, which means that they have no POD. Third, because this was a retrospective study in a single-center, generalization of the results may be limited.

## Conclusion

In conclusion, this study shows that even though TUR-BT is a minimally invasive surgery with a short intraoperative time, one in 12 patients experienced POD after TUR-BT under general anesthesia, and low body mass index, which is potentially modifiable, and older age were associated with POD.

## Supplementary Information

**Additional file 1: Supplemental Table 1.** The search strategies in Medline, Embase, PsycINFO, CINAHL, and Cochrane.

**Additional file 2: Supplemental Figure 1.** Preferred reporting items for systematic reviews and meta-analyses flow diagram of systematic search.

## Data Availability

They are available as a spreadsheet file upon reasonable request.

## Abbreviations

POD: Postoperative delirium
TUR-BT: Transurethral resection of bladder tumor
5-ALA: 5-Aminolevulinic acid
ICDSC: Intensive Care Delirium Screening Checklist
ASA-PS: American Society of Anesthesiologists-Physical Status
CI: Confidence interval
AUC: Area under the receiver operating characteristic curve
TUR: Transurethral resection
