# Supplementary material for: Incidence and factors associated with postoperative delirium in patients undergoing transurethral resection of bladder tumor
Source: JA Clin Rep. 2022 Jan 22;8:6. doi: 10.1186/s40981-022-00497-5 (PMC8783933; doi:10.1186/s40981-022-00497-5)
Supplement: Supplementary file 1 — Additional file 1: Supplemental Table 1. The search strategies in Medline, Embase, PsycINFO, CINAHL, and Cochrane. [file 40981_2022_497_MOESM1_ESM.docx]

Supplemental Table 1 The search strategies in Medline, Embase, PsycINFO, CINAHL, and Cochrane

Medline

1 (non-cardiac adj1 surger*).mp.

2 exp Urologic Surgical Procedures/

3 (Urolog* adj2 Surg* adj2 Procedur*).mp.

4 exp CYSTECTOMY/

5 Cystectom*.mp.

6 exp CYSTOSCOPY/

7 Cystoscop*.mp.

8 exp CYSTOTOMY/

9 Cystotom*.mp.

10 exp Kidney Transplantation/

11 (kidney* adj2 transplant*).mp.

12 exp NEPHRECTOMY/

13 Nephrectom*.mp.

14 exp NEPHROURETERECTOMY/

15 Nephroureterectom*.mp.

16 exp Nephrolithotomy, Percutaneous/

17 (Percutaneous adj1 Nephrolithotom*).mp.

18 exp NEPHROTOMY/

19 Nephrotom*.mp.

20 exp Nephrostomy, Percutaneous/

21 (Percutaneous adj2 Nephrostom*).mp.

22 exp URETEROSCOPY/

23 Ureteroscop*.mp.

24 exp Urinary Diversion/

25 (Urin* adj2 Diversion*).mp.

26 exp CYSTOSTOMY/

27 Cystostom*.mp.

28 exp URETEROSTOMY/

29 Ureterostom*.mp.

30 exp Circumcision, Male/

31 (male adj1 circumcis*).mp.

32 exp ORCHIECTOMY/

33 Orchiectom*.mp.

34 exp ORCHIOPEXY/

35 Orchiopex*.mp.

36 exp Penile Implantation/

37 (Peni* adj1 Implant*).mp.

38 exp PROSTATECTOMY/

39 Prostatectom*.mp.

40 exp VASECTOMY/

41 Vasectom*.mp.

42 exp VASOVASOSTOMY/

43 Vasovasostom*.mp.

44 exp Ureteral Obstruction/

45 pyeloplast*.mp.

46 (urethral adj2 stent*).mp.

47 (transurethral adj2 resection*).mp.

48 1 or 2 or 3 or 4 or 5 or 6 or 7 or 8 or 9 or 10 or 11 or 12 or 13 or 14 or 15 or 16 or 17 or 18 or 19 or 20 or 21 or 22 or 23 or 24 or 25 or 26 or 27 or 28 or 29 or 30 or 31 or 32 or 33 or 34 or 35 or 36 or 37 or 38 or 39 or 40 or 41 or 42 or 43 or 44 or 45 or 46 or 47

49 exp CONFUSION/

50 exp DELIRIUM/

51 exp Mental Processes/

52 exp Cognition Disorders/

53 (confus* or deliri*).mp.

54 49 or 50 or 51 or 52 or 53

55 48 and 54

56 exp Postoperative Period/

57 exp Postoperative Complications/

58 postop*.mp.

59 post-op*.mp.

60 56 or 57 or 58 or 59

61 55 and 60

62 limit 61 to ez=20200122-20210711

Embase

S1((non-cardiac n/1 surger*))

S2 (EMB.EXACT.EXPLODE("urologic surgery"))

S3 ((Urolog* n/1 Surg*))

S4 EMB.EXACT.EXPLODE("cystectomy")

S5 Cystectom*

S6 EMB.EXACT.EXPLODE("cystoscopy")

S7 Cystoscop*

S8 EMB.EXACT.EXPLODE("cystotomy")

S9 Cystotom*

S10 (EMB.EXACT.EXPLODE("kidney transplantation"))

S11 ((kidney* n/2 transplant*))

S12 EMB.EXACT.EXPLODE("nephrectomy")

S13 Nephrectom*

S14 EMB.EXACT.EXPLODE("nephroureterectomy")

S15 Nephroureterectom*

S16 ((Percutaneous n/1 Nephrolithotom*))

S17 EMB.EXACT.EXPLODE("nephrotomy")

S18 Nephrotom*

S19 (EMB.EXACT.EXPLODE("percutaneous nephrostomy"))

S20 ((Percutaneous n/2 Nephrostom*))

S21 EMB.EXACT.EXPLODE("ureteroscopy")

S22 Ureteroscop*

S23 (EMB.EXACT.EXPLODE("urinary diversion"))

S24 ((Urin* n/2 Diversion*))

S25 EMB.EXACT.EXPLODE("cystostomy")

S26 Cystostom*

S27 EMB.EXACT.EXPLODE("ureterostomy")

S28 Ureterostom*

S29 EMB.EXACT.EXPLODE("circumcision")

S30 ((male n/1 circumcis*))

S31 EMB.EXACT.EXPLODE("orchiectomy")

S32 Orchiectom*

S33 EMB.EXACT.EXPLODE("orchidopexy")

S34 (Orchiopex* OR Orchidopex*)

S35 (EMB.EXACT.EXPLODE("penile prosthesis implantation"))

S36 ((Peni* n/1 Implant*))

S37 EMB.EXACT.EXPLODE("prostatectomy")

S38 Prostatectom*

S39 EMB.EXACT.EXPLODE("vasectomy")

S40 Vasectom*

S41 EMB.EXACT.EXPLODE("vasovasostomy")

S42 Vasovasostom*

S43 (EMB.EXACT.EXPLODE("ureter obstruction"))

S44 pyeloplast*

S45 ((urethral n/2 stent*))

S46 ((transurethral n/2 resection*))

S47 (S1 or S2 or S3 or S4 or S5 or S6 or S7 or S8 or S9 or S10 or S11 or S12 or S13 or S14 or S15 or S16 or S17 or S18 or S19 or S20 or S21 or S22 or S23 or S24 or S25 or S26 or S27 or S28 or S29 or S30 or S31 or S32 or S33 or S34 or S35 or S36 or S37 or S38 or S39 or S40 or S41 or S42 or S43 or S44 or S45 or S46)

S48 EMB.EXACT.EXPLODE("confusion")

S49 EMB.EXACT.EXPLODE("delirium")

S50 ((confus* or deliri*))

S51 (EMB.EXACT.EXPLODE("postoperative period"))

S52 (EMB.EXACT.EXPLODE("postoperative complication"))

S53 postop*

S54 post-op*

S55 (S48 or S49 or S50)

S56 (S51 or S52 or S53 or S54)

S57 (S47 and S55 and S56)

S58 FAV(20200122-20210710)

S59 (S57 and S58)

PsycINFO

(((Year: [2020 TO 2021] OR TestYear: [2020 TO 2021]))) AND ((((((Any Field: (Cystectom*))) OR ((Any Field: (Cystoscop*))) OR ((Any Field: (Cystotom*))) OR (((Any Field: (kidney* NEAR/2 transplant*)))) OR ((Any Field: (Nephrectom*))) OR ((Any Field: (Nephroureterectom*))) OR (((Any Field: (Percutaneous NEAR/2 Nephrolithotom*)))) OR ((Any Field: (Nephrotom*))) OR (((Any Field: (Percutaneous NEAR/2 Nephrostom*)))) OR ((Any Field: (Ureteroscop*))) OR (((Any Field: (Urin* NEAR/2 Diversion*)))) OR ((Any Field: (Cystostom*))) OR ((Any Field: (Ureterostom*))) OR (((Any Field: (male NEAR/2 circumcis*)))) OR ((Any Field: (Orchiectom*))) OR ((Any Field: (Orchiopex*))) OR (((Any Field: (Peni* NEAR/2 Implant*)))) OR ((Any Field: (Prostatectom*))) OR ((Any Field: (Vasectom*))) OR ((Any Field: (Vasovasostom*))) OR ((Any Field: (pyeloplast*))) OR (((Any Field: (urethra* NEAR/2 stent*)))) OR (((Any Field: (transurethral NEAR/2 resection*)))))) OR ((((Any Field: (urolog* NEAR/2 surg*)))))) AND (((Index Terms: ("Postsurgical Complications"))) OR ((Any Field: (postop*)) OR (Any Field: (post-op*)))) AND (((Index Terms: ("Cognitive Processes"))) OR ((Index Terms: ("Cognitive Impairment"))) OR ((Index Terms: ("Delirium"))) OR ((Index Terms: ("Mental Confusion"))) OR ((Any Field: (confus*)) OR (Any Field: (deliri*))))

CINAHL

S1 (MH "Confusion+")

S2 (MH "Delirium")

S3 (MH "Mental Processes+")

S4 (MH "Cognition Disorders+")

S5 (confus* or deliri*)

S6 S1 OR S2 OR S3 OR S4 OR S5

S7 (MH "Postoperative Period")

S8 (MH “Postoperative Complications+”)

S9 postop*

S10 post-op*

S11 S7 OR S8 OR S9 OR S10

S12 (MH “Surgery, Urogenital+”)

S13 non-cardiac N1 surger*

S14 Urolog* N2 Surg* N2 Procedur*

S15 Cystectom* OR Cystoscop* OR Cystotom* OR (kidney* N2 transplant*) OR Nephrectom* OR Nephroureterectom* OR (Percutaneous N1 Nephrolithotom*) OR Nephrotom* OR (Percutaneous N2 Nephrostom*) OR Ureteroscop* OR (Urin* N2 Diversion*) OR Cystostom* OR Ureterostom* OR (male N1 circumcis*) OR Orchiectom* OR Orchiopex* OR (Peni* N1 Implant*) OR Prostatectom* OR Vasectom* OR Vasovasostom* OR pyeloplast* OR (urethral N2 stent*) OR (transurethral N2 resection*)

S16 S12 OR S13 OR S14 OR S15

S17 S6 AND S11 AND S16

S18 EM 20200122-20210711

S19 S17 AND S18

Cochrane

#1 [mh "Confusion"]

#2 [mh "Delirium"]

#3 [mh "Mental Processes"]

#4 [mh "Cognition Disorders"]

#5 confus* or deliri*

#6 #1 or #2 or #3 or #4 or #5

#7 [mh "Postoperative Period"]

#8 [mh "Postoperative Complications"]

#9 postop*

#10 post-op*

#11 #7 or #8 or #9 or #10

#12 [mh "Urologic Surgical Procedures"]

#13 non-cardiac near/1 surger*

#14 Urolog* near/2 Surg* near/2 Procedur*

#15 Cystectom* or Cystoscop* or Cystotom* or (kidney* near/2 transplant*) or Nephrectom* or Nephroureterectom* or (Percutaneous near/1 Nephrolithotom*) or Nephrotom* or (Percutaneous near/2 Nephrostom*) or Ureteroscop* or (Urin* near/2 Diversion*) or Cystostom* or Ureterostom* or (male near/1 circumcis*) or Orchiectom* or Orchiopex* or (Peni* near/1 Implant*) or Prostatectom* or Vasectom* or Vasovasostom* or pyeloplast* or (urethral near/2 stent*) or (transurethral near/2 resection*)

#16 #12 or #13 or #14 or #15

#17 #6 and #11 and #16

#18 #6 and #11 and #16 in Trials
