# Supplementary material for: Incidence and factors associated with postoperative delirium in patients undergoing transurethral resection of bladder tumor
Source: JA Clin Rep. 2022 Jan 22;8:6. doi: 10.1186/s40981-022-00497-5 (PMC8783933; doi:10.1186/s40981-022-00497-5)

Supplemental Figure 1 Preferred reporting items for systematic reviews and meta-analyses flow diagram of systematic search.

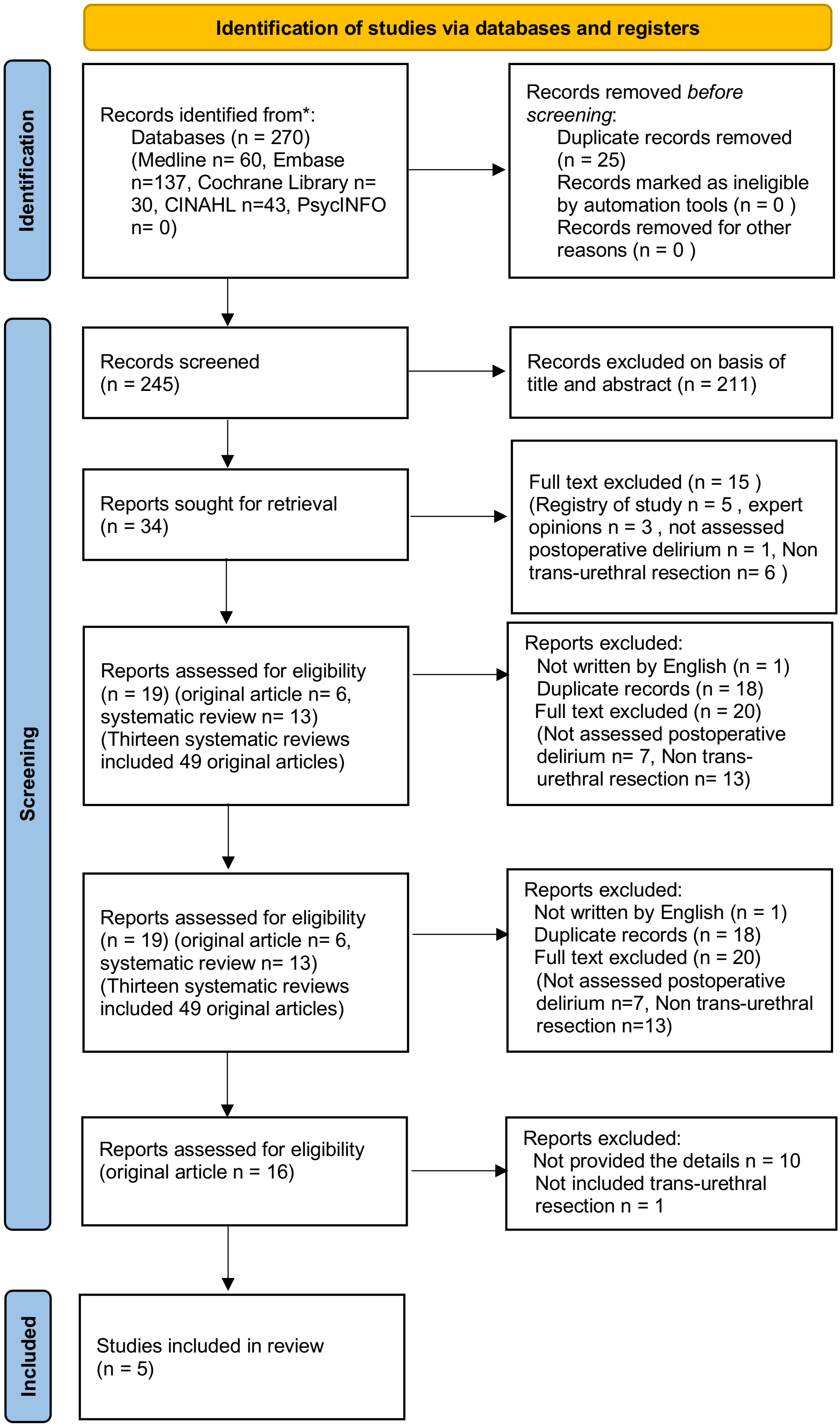

Supplement: Supplementary file 2 — Additional file 2: Supplemental Figure 1. Preferred reporting items for systematic reviews and meta-analyses flow diagram of systematic search. [file 40981_2022_497_MOESM2_ESM.pdf]
